# Evaluation and Comparison of Biological Cleaning Efficacy of Two Endofiles and Irrigants as Judged by Microbial Quantification in Primary Teeth — An *In Vivo* Study

**DOI:** 10.5005/jp-journals-10005-1013

**Published:** 2009-12-26

**Authors:** Iqbal Musani, Varun Goyal, Asha Singh, Chetan Bhat

**Affiliations:** 1 Professor, Department of Pedodontics and Preventive Dentistry, Dr DY Patil Dental College Hospital, Pune, Maharashtra India; 2 Postgraduate Student, Department of Pedodontics and Preventive Dentistry, Dr DY Patil Dental College Hospital, Pune Maharashtra, India; 3 Professor, Department of Pedodontics and Preventive Dentistry, Dr DY Patil Dental College Hospital, Pune, Maharashtra, India; 4 Senior Lecturer, Department of Pedodontics and Preventive Dentistry, Dr DY Patil Dental College Hospital, Pune, Maharashtra India

**Keywords:** Post and core, apexogenesis, reinforced tooth.

## Abstract

The endodontic triad comprises of cleaning and shaping, disinfection and obturation. Success of root canal therapy is majorly achieved by proper cleaning and shaping. However, elimination of bacterial contaminants as well as necrotic debris of the canals requires the adjunctive use of irrigants.

To achieve a satisfactory biological and mechanical preparation proper selection of endodontic instruments and irrigants is necessary. In this study we are comparing and evaluating cleaning efficacy of endofiles (K-files and handprotapers ) and root canal irrigants (sodium hypochlorite and chlorhexidine) by microbial quantification. Root canal samples were collected in autoclavable bottles containing transport media (nutrient broth) and samples were cultured in tryptose soya agar at incubation temperature of 37°C for 24-48 hours and colonies were counted with digital colony counter.

The significance of this study is to help the clinician select proper instrument and irrigant which minimize the failure rate of root canal treatment for the benefit of patients.

## INTRODUCTION

The specialty of endodontics has evolved and got revolutionized over the years. The modern endodontic specialty practice has little resemblance to the traditional endodontic practice. In the field of nonsurgical endodontics advancements have occurred in the techniques and materials been used.^12^ Root canal therapy (RCT) is the most common endodontic procedure been carried out today. In RCT each step determines the success of the successive step. The endodontic triad for successful root canal therapy comprises of cleaning and shaping, disinfection and obturation.

So to attain success a satisfactory biological and mechanical preparation with proper selection of endodontic irrigant and instrument is necessary. The cleaning and shaping process involves the removal of pulp cavity content and the consequent reduction of microorganism counts especially in nonvital teeth with periapical lesion. It is difficult to eliminate all the microorganisms and organic debris from the root canal without the use of the irrigants during preparation.

It has been shown that, when a negative microbiological culture is obtained from the root canal at the time of obturation, there is a 94% success rate. On the other hand, when the cultures are positive, and obturation is performed the success rate is reduced to 68%, which confirms the importance of sterile root canals for endodontic success.^11^

Endodontic treatment in primary teeth can be challenging and time consuming especially during canal preparation.

Significant alterations and complexities in root canal morphology of primary teeth demands improvement in the instrument design and irrigants to prevent undesirable complications and failures.

Over the years, many instruments with varying design made of different materials and with system oriented techniques were applied to achieve mechanical and biological objectives of the root canal treatment.

Until 1960, root canal instrument were produced of carbon steel which are now replaced by stainless steel instruments. Hatton et al (1928) said canal prepared with stainless steel instruments were only superficially cleaned and much of the pulp tissue was not removed, stainless steel has also been shown to create aberrations, probably as a result of the inherent stiffness of stainless steel, which is compounded by instrument design and shape.

In the modern endodontic practice various advances in the instruments such as changes involving metallurgy, instrument taper, and the way files are used in the root canals. Recently NiTi have been developed and are now widely used in endodontics as an efficient technique. The design and high flexibility of NiTi files allow instruments to closely follow the original root canal path especially in curved canals and the procedural errors such as ledges, over instrumentation and apical transportation are greatly reduced as well.

Numerous solutions have been used in endodontics to achieve desired success. Such as sodium hypochlorite which was introduced by walker in 1936. Till now it is the most popular root canal irrigant because of its germicidal potential and its ability to dissolve organic material however, sodium hypochlorite is irritant to periapical tissues.^1^ Chlorhexidine has been recognized as an effective antimicrobial agent. Its broad spectrum of antibacterial activity, substantivity and biocompatibility are some of the properties that justify its clinical use.^10^

So the purpose of this study is to help clinician not only in judicious selection of the root canal instrument, but as well as irrigant so that satisfactory cleaning and shaping can be achieved.

## AIMS AND OBJECTIVES

This Study aims to evaluate and compare biological cleaning efficacy of two endofiles (K-files and handprotaper) and irrigants (3% NaOCl and 2% chlorhexidine) by microbial quantification.

## MATERIAL AND METHODS

Forty childrens in the age group of 4-6 years of age who had reported to the Department of Pedodontics and Preventive dentistry at Dr DY Patil Dental college and Hospital, Pune with chief complaint of pain in lower back region of jaw.

### Criteria for Case Selection

1. H/O spontaneous pain.
2. Pain persists after relieving of aggregating factors and lingers for few minutes.
3. Restorable tooth structure.
4.
  - Clinically: Frank pulpal exposure
  - Pulpal status: Chronic symptomatic Pulpalgia with apical periodontitis
  - Pain on percussion: Positive.
5.
  - Pulp vitality test: Electric test - Positive
  - Thermal test - Positive.
6. Radiographic examination: Reveals involvement of pulp with apical periodontitis.
7. No evidence of fracture or trauma.

### Distribution of Subjects

**Figure:**
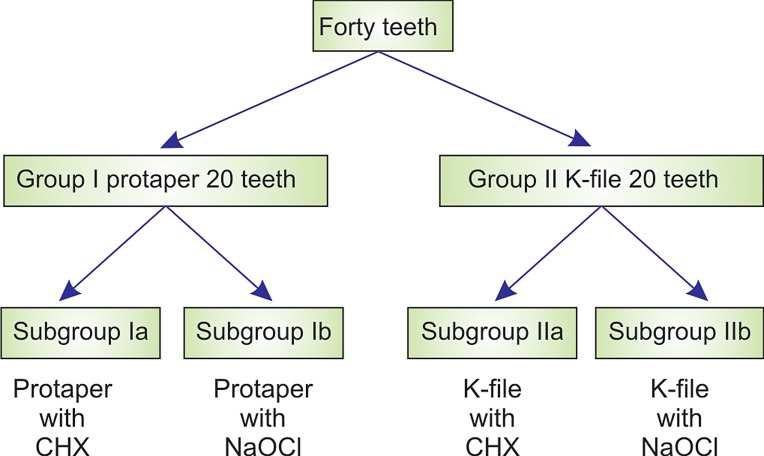


**Fig. 1.**
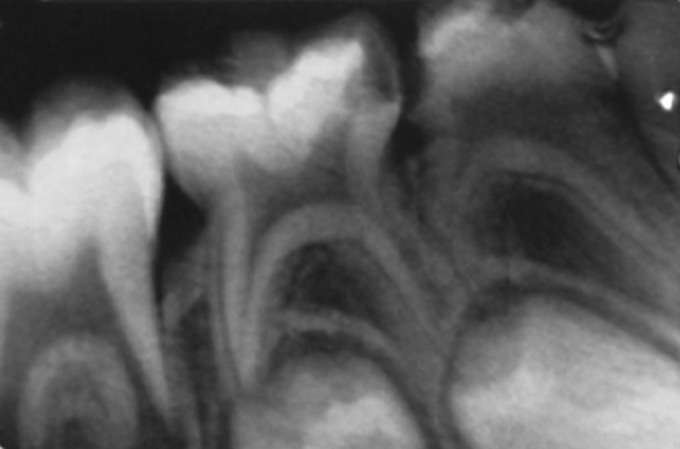
All patients were radiographed for accurate diagnosis

At the start of study, the children were subjected to a clinical and radiographic examination and the medical and dental history of child were taken. Pulpectomy indicated cases were chosen for the study (Fig. 1).

Under strict aseptic conditions, the procedural tooth was anesthetized and isolated with rubber dam. The tooth and adjacent rubber dam were disinfected with a tincture of iodine.

**Fig. 2.**
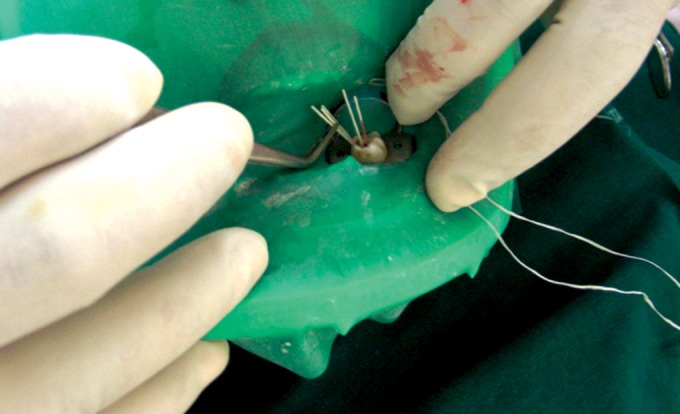
Strict asepsis protocols being followed for the cases

**Fig. 3.**
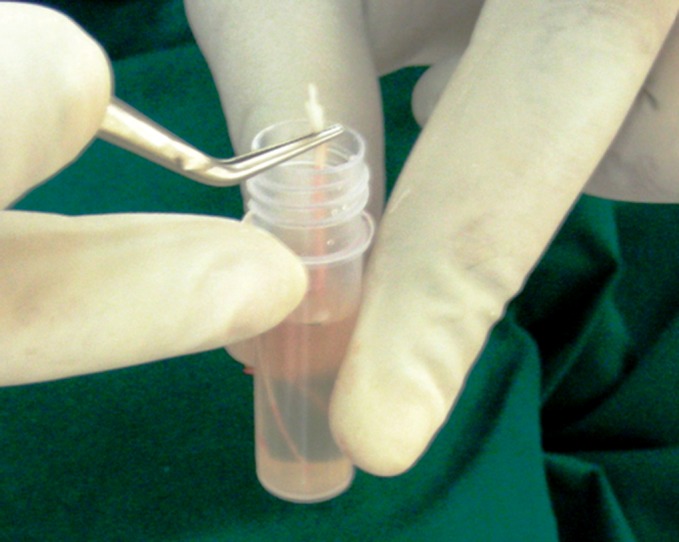
Root canal contents were obtained and transferred into the sterile container containing 5% nutrient broth and sealing the bottle tightly

Endodontic access was achieved with a sterile high speed carbide bur. If rubber dam isolation could not be maintained during pulpectomy procedure, the case was eliminated from the study. After access was achieved, the tooth and adjacent rubber dam were once again disinfected with 30% hydrogen peroxide and a tincture of iodine (Fig. 2). After the tincture had dried, the tooth surface was swabbed with a 5% sodium thiosulfate solution to inactivate the iodine tincture so that remnants of it would not influence the bacteriologic sampling. On gaining access to the pulp, a sterile broach was inserted into the root canal up to the apical foramina and root canal content were obtained and transferred into the sterile container containing 5% nutrient broth and sealing the bottle tightly for onward transfer to the microbiological laboratory of DY Patil Medical College, Pune for microbial quantification. Then working length determination was done by radiographic method (Fig. 3).

In subgroup Ia, 10 samples were prepared with hand protaper under copious irrigation with 2% CHX. The protaper technique for deciduous teeth differs from permanent teeth to prevent lateral perforation of the canals. The Sx file was inserted into the canal to about 3 mm beyond the root canal orifice with a slight (buccolingual) brushing motion to remove any overlying dentin and to improve straight line access. The S2-file was then inserted into the canal and taken to the working length as previously determined.^14^

In subgroup Ib, the samples were prepared with hand protaper but under copious irrigation with 3% NaOCl.

In subgroup IIa, 10 samples were prepared with K-files under copious irrigation with 2% CHX the apical preparation was done till 35 no. K-file. In subgroup IIb, also the samples were prepared with K-files but under copious irrigation with 3% NaOCl.

**Fig. 4.**
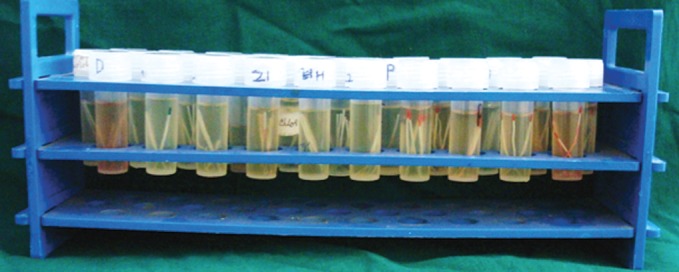
All samples stored for further testing

After cleaning and shaping, the samples were again collected with the help of 15 no. sterile absorbent points and transferred to the laboratory in nutrient broth for microbial examination (Fig. 4).

### Microbiological Examination

**Fig. 5.**
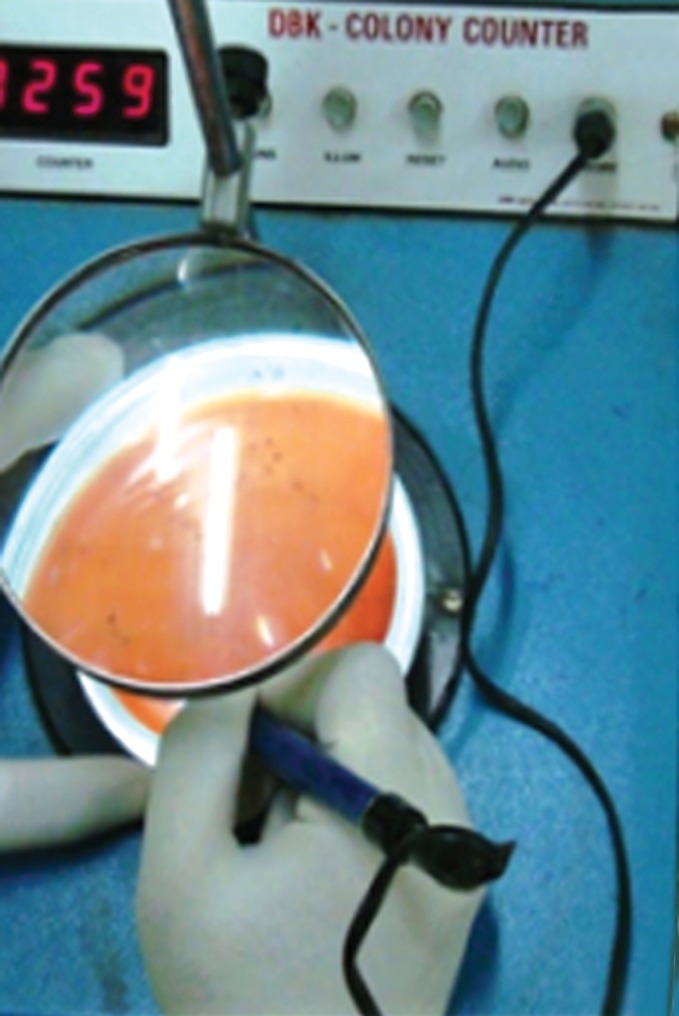
Digital colony counter

The vials containing samples were agitated for 30 seconds on a vortex at power setting 4 before aliquot disbursement. Sample dilutions of 2.3 logs were accomplished, and 49/µl of the sample were delivered to a plate by means of a Model D Spiral Plater. Plates were inoculate with tryptose soya agar with added 5% of human blood. They were incubated at temperature of 37°C for 1-3 days. Colony characteristics were noted in case of any growth (Groups Ia, Ib, IIa and IIb). Colony counting in this study was carried out with digital colony counter (Fig. 5).

## RESULTS

The data was analyzed statistically using Student’s Paired ‘t’ test. Pre- and Postmicrobial values of Group Ia, Ib, IIa, IIb were calculated.

Mean values and SD of postmicrobial count of Group Ia (Protaper with chlorhexidine), Group Ib (Protaper with NaOCl), Group IIa (K-file with chlorhexidine) and Group IIb (K-file with NaOCl) are listed in Table 1. The lowest Post microbial count was obtained in group (Ia < IIa < Ib < IIb).

## DISCUSSION

The cleaning and shaping of root canals are relevant phases in root canal therapy. Schilder described five design objectives:^13^

**Table:** TABLE 1: Postmicrobial mean values with standared deviation

*Groups*		*No. of*		*Mean*		*Std.*		*Minimum*		*Maximum*
		*samples*				*deviation*				
Ia		10		462.50		232.80		250.00		750.00
Ib		10		5175.00		5369.27		150.00		10350.00
IIa		10		4350.00		2821.64		2200.00		8450.00
IIb		10		8350.00		13470.33		300.00		28500.00
Total		40								

**Figure:**
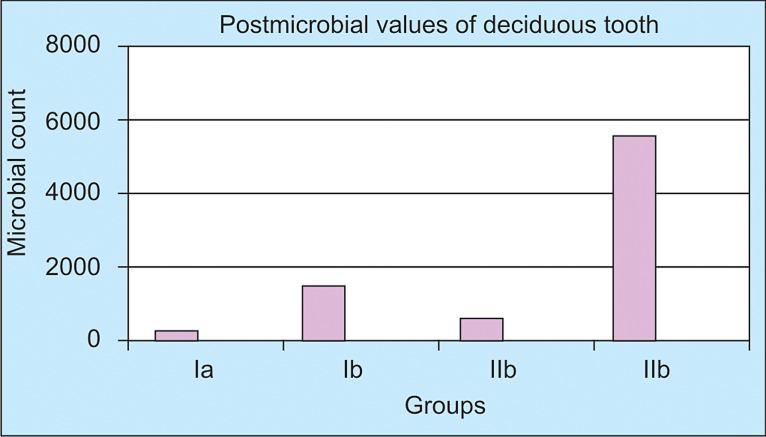

1. Continuously tapering funnel from the apex to the access cavity.
2. Cross-sectional diameter should be narrower at every point apically.
3. The root canal preparation should flow with the shape of the original canal.
4. The apical foramen should remain in its original position.
5. The apical opening should be kept as small as practical.

And four biologic objectives:

1. Confinement of instrumentation to the roots themselves.
2. No forcing of necrotic debris beyond the foramen.
3. Removal of all tissue from the root canal space.
4. Creation of sufficient space for intracanal medicaments.

Cleaning of root canals may be done by mechanical and/or chemical means. The literature has suggested the chemical means as the effective way of cleaning of root canals in primary teeth, as the mechanical means is considered to be injurious to the succedaneous tooth bud in cases of over instrumentation of root canals beyond the apex.^6^

But the recent literature gives a different picture in which it is been stated that the value of mechanical cleaning cannot be overlooked and the chemomechanical means is the most effective way to clean the root canals in primary and permanent teeth. The chemical means are used in conjunction with the limited mechanical debridement, to disinfect and remove necrotic material within the somewhat inaccessible canals, rather than "shaping" of the canals.^15^

**Group Ia(A):**
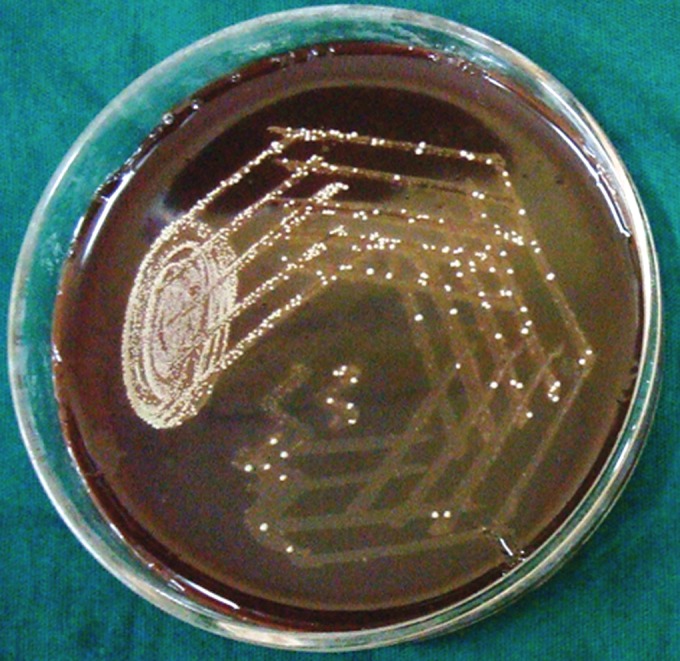
Petri dish showing microbiological colony prior to cleaning and shaping

**Group Ia(B):**
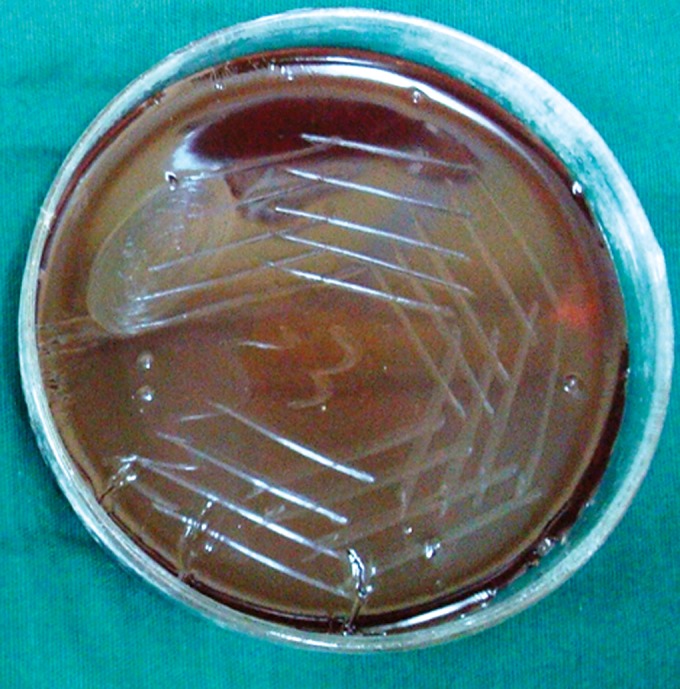
Petri dish showing microbiological colony after cleaning and shaping

**Group Ib(A):**
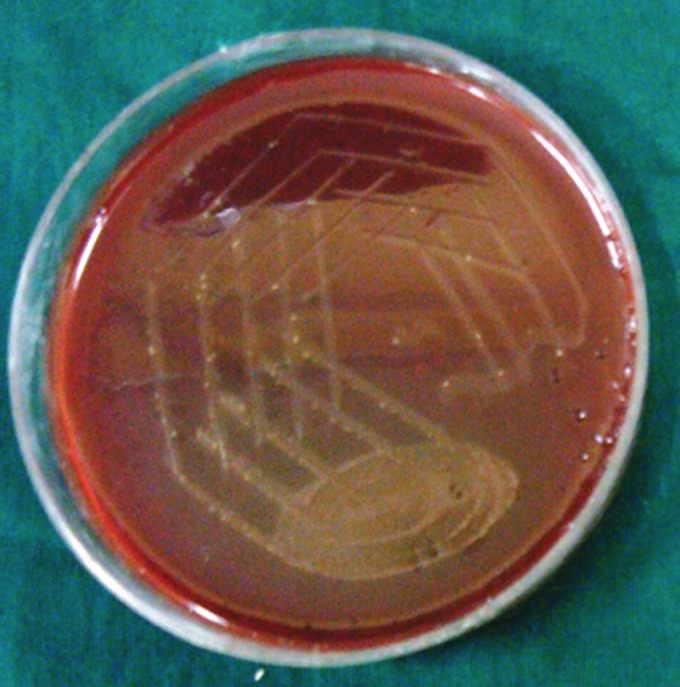
Petri dish showing microbiological colony prior to cleaning and shaping

**Group Ib(B):**
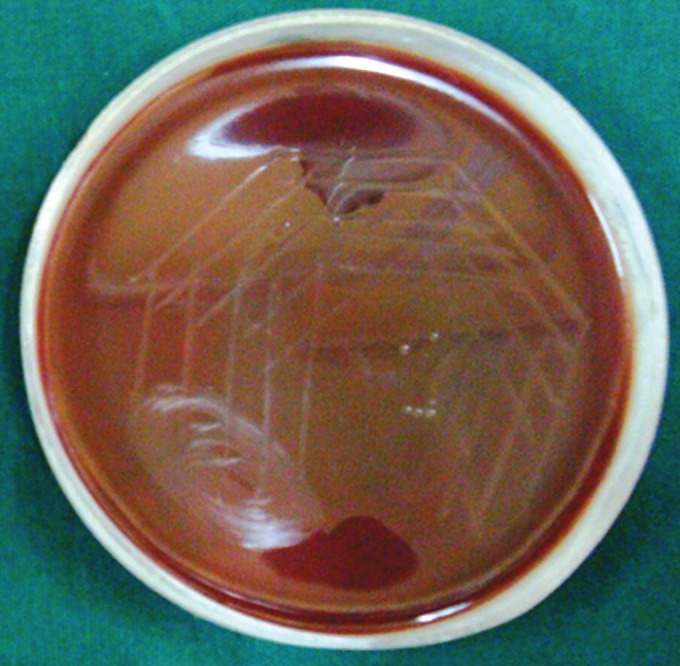
Petri dish showing microbiological colony after cleaning and shaping

**Group IIa(A):**
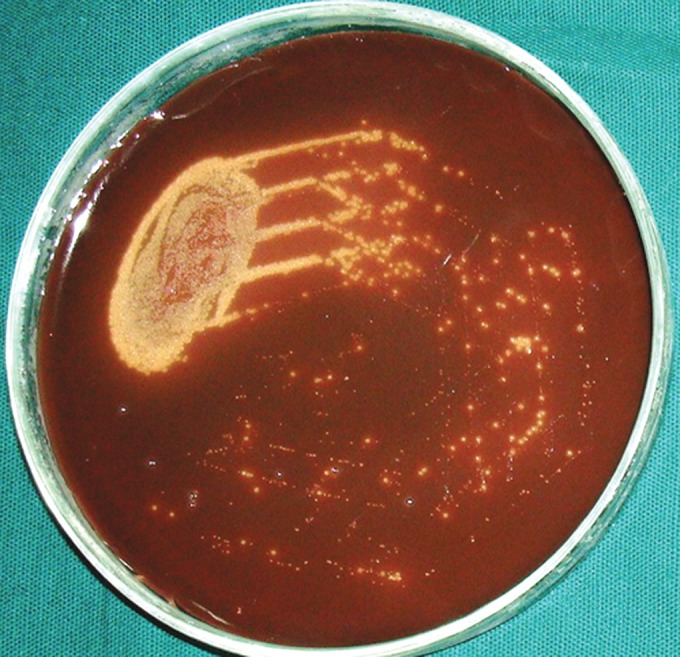
Petri dish showing microbiological colony prior to cleaning and shaping

**Group IIa(B):**
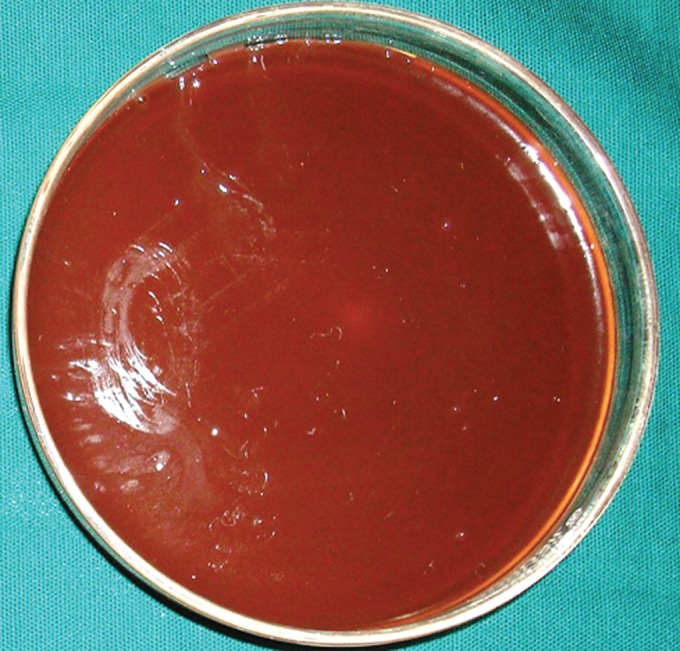
Petri dish showing microbiological colony after cleaning and shaping

**Group IIb(A):**
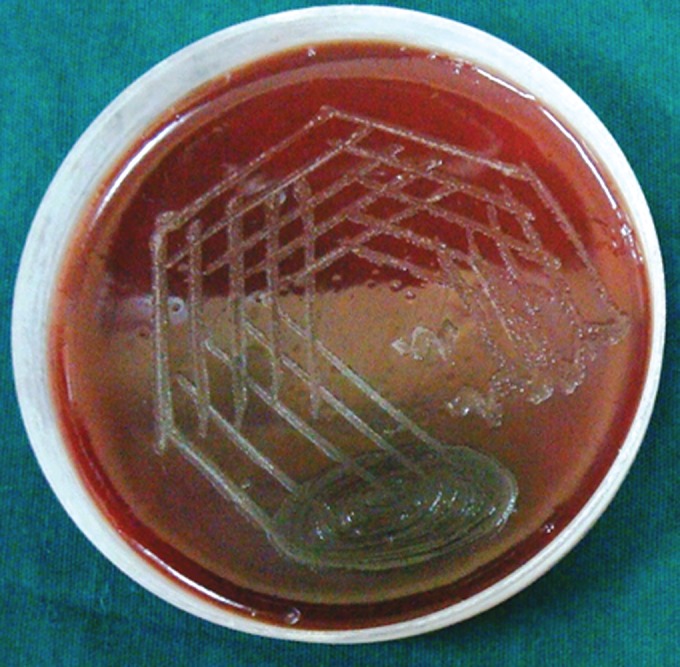
Petri dish showing microbiological colony prior to cleaning and shaping

**Group IIb(B):**
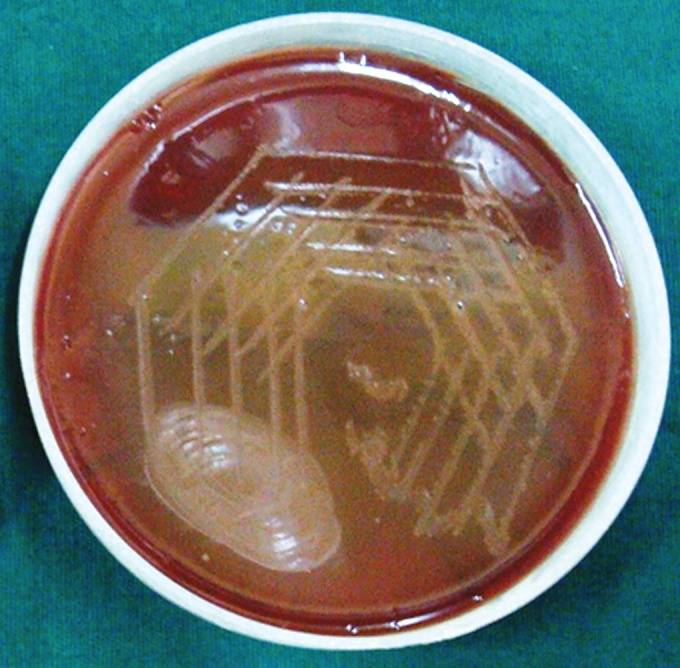
Petri dish showing microbiological colony after cleaning and shaping

Mechanical cleaning involve; enlarging the canal wall by instruments and mechanical flushing action of the irrigant solution. It is believed that thorough debridement before sealing the root canal system is the key to long-term success of endodontic therapy.^2^ Culture obtained before obturation is a major factor to determine the long-term prognosis of the tooth. Thus, the difference in canal cleanliness was evaluated by microbiological examination. The volume and method used for irrigation were controlled in all groups. Chemomechanical preparation is one of the most important phase of endodontic therapy. The best known and most common instruments and irrigants are K-files and NaOCl. The established clinical efficacy of K-file results from its square blank, tight spiral and cutting angle whereas NaOCl is popular because of its ability to dissolve necrotic tissue and organic remnants and its antimicrobial activity. On the other hand, the inherent stiffness of stainless steel instruments create aberration in the canal and NaOCl also has certain adverse effects such as corrosion of endodontic instruments, toxicity, odor, ineffectiveness against some microorganisms when used in low concentrations and not differentiating between necrotic and vital tissues when in contact with apical and periapical tissues. Therefore attempts have been made to develop new instruments and irrigating solutions for thorough debridement.

In the present study Protaper, K-files, NaOCl and Chlorhexidine were compared. The result indicates better cleaning with Group Ia (Protaper and Chlorhexidine) as compared to other groups. The protaper files have a continuously changing helical angle and pitch, this balancing of pitch and helical angle optimizes its debris removing capacity.^7^

Research evaluating canal cleanliness has shown that preparation need to be taper at least 0.08 mm/mm to 0.10 mm/mm to ensure that a sufficient volume of irrigant can efficaciously circulate into the canal anatomy (Allison et al,^3^ Machtou et al,^4^ McGreevey^8^).

Parson and associates^5^ in 1980 first evaluated the antimicrobial efficacy of chlorhexidine and concluded that chlorhexidine was a potent antimicrobial agent and may serve as an effective endodontic irrigant.

Studies by Validaty et al and Yesilsoy et al^9^ have shown that chlorhexidine gluconate to be an effective antimicrobial agent as sodium hypochlorite *in vitro*. Further studies by Jeanosonne and White shown that 2% chlorhexidine resulted in fewer positive postirrigant cultures than did 5.25% NaOCl. The present study also showed that the Group Ia, IIa have significantly higher antimicrobial action than Group Ib, IIb. There was a significant reduction in the total viable count of microorganisms with both the irrigant. These results suggest that the endodontic treatment is dependent on the association of a chemical and mechanical approaches and that root canal enlargement improves bacterial reduction probably because the irrigation has more access to the apical third.

The result of the present study and those from previous *in vitro* and *in vivo* studies suggest that 2% chlorhexidine may be used as an irrigating solution in root canals due to its superior intracanal antimicrobial action.

The limitation of this study was the inability to standardize the preoperative positive culture in all the 4 groups studied. The substantive action of chlorhexidine due to its property to adsorb to anionic substrate was also not considered in this study. Therefore, a more extensive research with a definitive data distribution and use of parametric test is required to evaluate the two instruments and irrigants in future.

## CONCLUSION

Canal debridement is challenging considering the morphological complexities of root canals and the nature of microbial infection. So to combat with these challenges an efficient and effective chemomechanical preparation of root canal is mandatory.
